# Homocysteine induces podocyte apoptosis by regulating miR‐1929‐5p expression through *c‐Myc*, DNMT1 and EZH2

**DOI:** 10.1002/1878-0261.13032

**Published:** 2021-07-19

**Authors:** Lin Xie, Shengchao Ma, Ning Ding, Yanhua Wang, Guanjun Lu, Lingbo Xu, Qingqing Wang, Kun Liu, Yuzheng Jie, Hui Zhang, Anning Yang, Yujing Gao, Huiping Zhang, Yideng Jiang

**Affiliations:** ^1^ School of Basic Medical Sciences Ningxia Medical University Yinchuan China; ^2^ NHC Key Laboratory of Metabolic Cardiovascular Diseases Research Ningxia Medical University Yinchuan China; ^3^ Ningxia Key Laboratory of Vascular Injury and Repair Research Ningxia Medical University Yinchuan China; ^4^ Department of Clinical Medicine Ningxia Medical University Yinchuan China; ^5^ Prenatal Diagnosis Center of General Hospital Ningxia Medical University Yinchuan China

**Keywords:** *c‐Myc*, DNMT1, EZH2, homocysteine, kidney, miR‐1929‐5p

## Abstract

Chronic kidney disease (CKD) is a common and complex disease in kidneys which has been associated with an increased risk of renal cell carcinoma. Elevated homocysteine (Hcy) levels are known to influence the development and progression of CKD by regulating podocyte injury and apoptosis. To investigate the molecular mechanisms triggered in podocytes by Hcy, we used *cbs^+/−^* mice and observed that higher Hcy levels increased the apoptosis rate of podocytes with accompanying glomerular damage. Hcy‐induced podocyte injury and apoptosis in *cbs^+/−^* mice was regulated by inhibition of microRNA (miR)‐1929‐5p expression. Overexpression of miR‐1929‐5p in podocytes inhibited apoptosis by upregulating Bcl‐2. Furthermore, the expression of miR‐1929‐5p was regulated by epigenetic modifications of its promoter. Hcy upregulated DNA methyltransferase 1 (DNMT1) and enhancer of zeste homolog 2 (EZH2) levels, resulting in increased DNA methylation and H3K27me3 levels on the miR‐1929‐5p promoter. Additionally, we observed that *c‐Myc* recruited DNMT1 and EZH2 to the miR‐1929‐5p promoter and suppressed the expression of miR‐1929‐5p. In summary, we demonstrated that Hcy promotes podocyte apoptosis through the regulation of the epigenetic modifiers DNMT1 and EZH2, which are recruited by *c‐Myc* to the promoter of miR‐1929‐5p to silence miR‐1929‐5p expression.

## Graphical abstract

Chronic kidney disease is a common and complex disease of the kidneys, associated with an increased risk of renal cell carcinoma. In the *cbs^+/−^* mouse model, we observed that homocysteine (Hcy) induced podocyte apoptosis by regulating miR‐1929‐5p expression. Hcy‐mediated upregulation of DNMT1 and EZH2 led to epigenetic miR‐1929‐5p silencing, following *c‐Myc*‐dependent recruitment of DNMT1 and EZH2 to the miR‐1929‐5p promoter. Inhibition of miR‐1929‐5p expression induced apoptosis in podocytes and led to kidney injury.

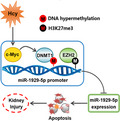

**Abbreviations**
AZC: 5‐azacytidine
BUN: blood urea nitrogen
*Cbs*
: cystathionine beta‐synthase
CKD: chronic kidney disease
Co‐IP: co‐immunoprecipitation
Cr: creatinine
CytC: cytochrome‐*c*
DAPI: 4′, 6‐diamidino‐2‐phenylindole
DNMT1: DNA methyltransferase 1
DNMT: DNA methyltransferase
dUTP: 2′-deoxyuridine 5′-triphosphate
EPZ: EZH2 specific inhibitor, EPZ005687
EZH2: enhancer of zeste homolog 2
GO: gene ontology
Hcy: homocysteine
HHcy: hyperhomocysteinemia
M.SssI: SssI DNA methylases
Mut: mutant type
nMS-PCR: nested methylation-specific-polymerase chain reaction
PAS: periodic acid-Schiff
qRT-PCR: quantitative real-time polymerase chain reaction
RCC: renal cell carcinoma
RIP: RNA-binding protein immunoprecipitation
RNA-seq: RNA-sequencing
SAM: *S*-adenosylmethionine
TEM: transmission electron microscopy
TUNEL: terminal deoxynucleotidyl transferase-mediated deoxyuridine 5-triphosphate nick end labeling
WT: wild type;TSS, transcription start site

## 1 Introduction

Renal cell carcinoma (RCC) is the most common pathology subtype of kidney cancer in the urinary system [1]. Studies have shown that chronic kidney disease (CKD) is a risk factor for RCC, and the increased morbidity and mortality of RCC due to CKD has recently been intensively discussed and evaluated [2]. An elevated level of the homocysteine (Hcy), hyperhomocysteinemia (HHcy), is connected with CKD and further contributes to kidney damage [3, 4]. As a unique glomerular epithelial cell involved in glomerular protein filtration, more and more experiments have highlighted the importance of podocytes in the development of CKD. Podocyte injury as a result of cell detachment and apoptosis has been described to be an early event in CKD [5, 6]. Furthermore, a recent report demonstrated that the apoptosis is a triggering mechanism leading to podocyte injury and glomerular sclerosis during HHcy [7]. Nevertheless, the mechanism of podocyte apoptosis initiation and the following glomerular injury during HHcy is not fully elucidated.

MicroRNA (miRNA) is a class of endogenous small non‐coding RNA (18–25 nt in length) that can regulate gene expression by binding to the 3′‐UTR of messenger RNA (mRNA) and control of various biological processes, such as differentiation, proliferation, migration and apoptosis [8, 9]. Recently, deep insight into kidney diseases revealed that miRNA play pivotal role in modulation of genes involved in glomerular diseases [10, 11, 12, 13]. But there is no miRNA marker available for clinical use and the underlying mechanisms of miRNA in podocyte apoptosis induced by Hcy remain largely unknown. Hence, the use of deregulated miRNA as prognostic markers and molecular therapeutic targets for glomerular diseases has triggered considerable interest.

Hcy is a methyl group carrier that affects the epigenetic regulation of gene expression primarily through the interference of methyl group transferring metabolism, in which a methyl group is transferred to macromolecules such as DNA and histone *in vivo*. This process is associated with epigenetic changes including DNA methylation and histone methylation [14, 15]. More importantly, epigenetic modifications do not work independently, they regulate gene expression through synergistic or antagonistic interactions [16]. Furthermore, Hcy transferred methyl group to DNA or histones, which prevent DNA or histones from recognizing and binding to transcription factors at gene promoters [17]. The oncogene *c‐Myc* encodes a conserved basic helix‐loop‐helix leucine zipper transcription factor and is known to regulate miRNA expression at the transcriptional level by binding to a conserved E‐box (CACGTG) [18]. In particular, *c‐Myc* preferentially associates with promoters enriched for euchromatic marks, including di‐ or tri‐ methylation of histone H3 lysine 27 or lysine 79 [19]. Thus, a comprehensive understanding of this dynamic interplay will set the stage for the discovery of pan‐cellular transcription factor regulatory strategies to predict kidney diseases risk and therapy response.

In this study, by studying miRNA in Hcy‐treated podocytes, we found that the cooperation of DNA hypermethylation and H3K27me3 that promotes the binding of *c‐Myc* to miR‐1929‐5p promoter, which in turn inhibited miR‐1929‐5p expression and promoted podocyte apoptosis. Our results provide novel insights into the molecular mechanism underlying Hcy‐induced podocyte apoptosis.

## 2 Materials and methods

### 2.1 Animals

Cystathionine beta‐synthase (*cbs*) heterozygous knockout (*cbs^+/−^*) mice (8–10 weeks of age) purchased from Jackson Laboratory (Bar Harbor, ME, USA) were maintained in a specific pathogen‐free environment. Since *cbs* homozygote knockout (*cbs^−/−^*) mice have a short lifespan and die of liver failure before weaning, *cbs^+/+^* and *cbs^+/−^* mice were used for all experiments. The male *cbs^+/−^* and *cbs^+/+^* mice were fed with chow diet plus 2.0% methionine to induce HHcy for 8 weeks. All experimental animal procedures were performed according to guidelines approved by the Institutional Animal Care and Use Committee at the Ningxia medical university Laboratory Animal Center (ethics approval number is NYDWZX‐2018‐083).

### 2.2 Cell culture and treatment

Conditionally immortalized mouse podocyte (MPC‐5) and HEK293 cells were cultured in the Dulbecco’s modified Eagle’s medium (Gibco, New York, NY, USA) supplemented with 10% FBS, 1% pen‐strep‐glutamine (Solarbio, Beijing, China) in a humidified atmosphere of 5% CO_2_ at 37 °C. Podocytes treated with 0 and 80 μm Hcy were used as the Control group and Hcy group, respectively. For transfection experiments, when the cell confluence reached 70%, miR‐1929‐5p mimic, miR‐1929‐5p inhibitor (Gene Pharma, Shanghai, China), Ad‐DNMT1 and Ad‐*c‐Myc* (Hanbio Biotechnology, Shanghai, China) were used for transfection, respectively. The Control cells were transfected with miRNA negative control (miNC) and Ad‐GFP. The specific small interfering RNA (siRNA) for *c‐Myc*, EZH2 and DNMT1 were purchased from Gene Pharma (Shanghai, China).

### 2.3 Plasmid construction

The 2200‐bp promoter region of miR‐1929‐5p was amplified by PCR using mouse genomic DNA as template followed by insertion into pGL3‐basic vector to produce pGL3‐miR‐1929‐5p‐promoter (Yingbio Technology, Shanghai, China). The wild‐type (WT) and mutated (Mut) Bcl‐2 cDNA were subcloned into the pcDNA3.1 vector to produce pcDNA‐Bcl‐2‐WT and pcDNA‐Bcl‐2‐Mut. Lipofectamine 2000 (Invitrogen, Carlsbad, CA, USA) was used for transient transfection according to the manufacturer’s instructions.

### 2.4 Transmission electron microscopy

The kidney tissues were cut into pieces (1 × 1 × 5 mm) and fixed with glutaraldehyde in 0.1 m PBS (pH 7.2) at 4 °C for 2 h. After washing with PBS, tissues were post‐fixed in the 1% osmium tetroxide for 1 h followed by dehydration with 30–100% ethanol gradient. The tissue was then embedded and sectioned with a diamond knife, stained with 1% uranyl acetate and 1% lead citrate, and finally examined with transmission electron microscope (Olympus, Tokyo, Japan).

### 2.5 Periodic acid‐Schiff staining

The sections were pretreated with periodic acid and then rinsed slowly in RNase‐free distilled water. After staining with Schiff’s solution for 10 min in the dark and counterstaining with hematoxylin, the sections were dipped in 1% hydrochloric alcohol six times, dehydrated with 30–100% graded alcohol and immersed in xylene. Finally, images were acquired using light microscopy (Leica, Heidelberg, Germany).

### 2.6 Cell viability and actin cytoskeleton

Total cell number was determined by nuclei staining with Nuclei Dye that can stain the nuclei of both live and dead cells. The percentage of dead cells was detected based on membrane permeability using the Dead Dye. Viable cells were also stained with the Viable Dye (Roche, Indianapolis, IN, USA). F‐actin was stained using the reagent of phalloidin‐iFluor™ 488 Conjugate (AAT Bioquest, Sunnyvale, CA, USA), the podocytes were permeabilized for 5 min with PBS containing 0.1% Triton X‐100 and then counterstained with 5 μg·mL^−1^ of 4′, 6‐diamidino‐2‐phenylindole (DAPI). Fluorescent images were acquired by a confocal microscope (Olympus).

### 2.7 Flow cytometric analysis

Podocytes were examined using a commercial PE Annexin V Apoptosis Detection Kit I (BD Bioscience Pharmingen, San Diego, CA, USA) and analyzed by flow cytometer (BD Bioscience, San Diego, CA, USA) according to the manufacturer’s instruction.

### 2.8 TUNEL assay

Apoptosis of kidney tissues was detected by terminal deoxynucleotidyl transferase‐mediated deoxyuridine 5‐triphosphate nick end labeling (TUNEL) using a fluorescein‐based kit (Roche) according to the manufacturer’s instructions. Briefly, frozen sections were fixed with 4% paraformaldehyde and digested with proteinase K (20 μg·mL^−1^) for 15 min. After washing with PBS for three times, the sections were incubated with TdT enzyme and 2′‐deoxyuridine 5′‐triphosphate (dqaUTP) mixture at 37 °C for 1 h followed by counterstained with DAPI. Fluorescent images were acquired by a confocal microscopy (Olympus). The TUNEL index was determined by calculating the percentage of TUNEL positive to DAPI‐labeled cells.

### 2.9 Western blotting analysis

Kidney tissues or podocytes were lysed in a lysis buffer (KeyGene, Shanghai, China) supplemented with the PMSF (KeyGene) at 4 °C for 30 min. After separation by 12% SDS/PAGE, the proteins were transferred to polyvinylidene difluoride membranes (Millipore, Boston, MA, USA) and blocked overnight using 5% nonfat dry milk in Tris‐buffered saline with 0.1% Tween 20. The membranes were probed with antibodies to Bax, Bcl‐2, caspase‐12, H3K27me1, 2, 3, EZH2, *c‐Myc*, DNMT1 (1 : 1000 dilution; Abcam, Cambridge, MA, USA) and β‐actin (1 : 1000 dilution; Zhongshan Biotech, Beijing, China). Protein signals were visualized using horseradish peroxidase‐conjugated secondary antibodies and enhanced chemiluminescence solution (KeyGene). All samples were derived at the same time and processed in parallel.

### 2.10 Immunofluorescence staining

The sections were permeabilized with PBS containing 0.2% Triton X‐100 followed by blocking with 10% goat serum for 1 h, and then incubation with primary antibodies at 4 °C overnight. After washing with PBS, the sections were incubated with a fluorescein isothiocyanate (FITC)‐conjugated and tetramethylrhodamine isothiocyanate‐conjugated secondary antibody (Abcam) for 1 h at 37 °C and counterstained with DAPI. The fluorescent images were collected by confocal microscope (Olympus). The individual fluorescent channel images were converted to 8‐bit grayscale, and the colocalization analysis was performed using coloc2 plug‐in for imagej (National Institutes of Health, Bethesda, MD, USA).

### 2.11 Quantitative real‐time polymerase chain reaction

Total RNA was extracted from kidney tissues or podocytes using the RNA isolation kit (Invitrogen) according to the manufacturer’s protocol. For miRNA, Bulge‐loop miRNA quantitative real‐time polymerase chain reaction (qRT‐PCR) primer specific for miR‐1929‐5p was designed, including one RT primer and a pair of qPCR primers (RiboBio, Guangzhou, China). For mRNA, the primers were designed by Sangon Biotech (Shanghai, China) and the primer sequences are listed in Table S1. Reverse transcription was then performed by First‐Strand cDNA Synthesis Kit (Invitrogen). The real‐time PCR was carried out by applying an FTC3000 real‐time PCR detection system (Funglyn Biotech Inc., Toronto, ON, Canada). Each sample was performed in triplicate and the data normalized using U6 or GAPDH as the internal calibrator.

### 2.12 Dual‐luciferase reporter assay

The wild type (WT) and mutant type (Mut) of miR‐1929‐5p promoter (from −2000 to +200) and Bcl‐2 3′‐UTR region were cloned to pGL3‐Basic luciferase vector (Promega, Madison, WI, USA). The Mut of miR‐1929‐5p promoter and Bcl‐2 3′‐UTR region were generated by inducing point mutation to the key nucleotides in binding motif using the Fast Mutagenesis System Kit (TransGen Biotech, Beijing, China) according to the manufacturer’s protocol. Finally, the dual‐luciferase reporter assay plasmids were used to transfect HEK293 cells and determine the relative luciferase activity. The renilla luciferase activity was used as the internal control for transfection efficiency.

### 2.13 Nested methylation‐specific‐polymerase chain reaction

The isolated genomic DNA was bisulfite‐modified using EZ DNA Methylation‐Gold™ kit (Zymo Research, Irvine, CA, USA). Nested methylation‐specific‐polymerase chain reaction (nMS‐PCR) consists two‐step PCR amplifications that are used for the detection of miR‐1929‐5p promoter methylation levels. The first step of nMS‐PCR uses an outer primer pair set that does not contain any CpGs. The second step of PCR was carried out with a methylation primer and an unmethylated primer. The primers used for the nMS‐PCR assays are listed in Table S2. The PCR products were separated by 2% agarose gel containing ethidium bromide and visualized with ultraviolet light. Methylation was calculated using the formula:

Methylation%=methylation/(methylation+unmethylation)×100%.

### 2.14 MassARRAY methylation analysis

The isolated genomic DNA from podocytes was bisulfite‐converted with the EpiTect Bisulfite Kit (Qiagen, Beijing, China) according to the manufacturer’s instruction. We designed primers for the miR‐1929‐5p promoter and selected amplicon to cover the promoter region with the most CpG sites. For each sample, at least three PCR product clones were randomly selected for DNA sequencing and following methylation analysis by methtools (Biomiao Biological Technology, Beijing, China). The mass spectra were collected using a MassARRAY Compact MALDI‐TOF (Biomiao Biological Technology, Beijing, China) and the methylation ratios of the spectra were generated by the epityper software (Sequenom, CA, USA).

### 2.15 Chromatin immunoprecipitation assay

Chromatin immunoprecipitation (ChIP) assays were performed according to the manufacturer’s instructions (Millipore, catalog no. 17‐371). Anti‐H3K27me3, anti‐EZH2 and anti‐*c‐Myc* (Abcam) were used for ChIP. qPCR primers were designed to detect the proximal promoter region of miR‐1929‐5p by RT‐PCR as described above; forward primer: 5′‐CGCTGCTGGGCGCTCTTCCTGTCTC‐3′ and reverse primer: 5′‐AGAGTGGGAGCGGGTCAT‐3′. IgG was used as a negative control to measure nonspecific backgrounds in immunoprecipitation.

### 2.16 RNA‐binding protein immunoprecipitation assay

The RNA‐binding protein immunoprecipitation (RIP) assay was conducted using an EZ‐Magna RIP Kit (Millipore, catalog no. 17‐701) following the manufacturer’s instructions. Cell lysates were incubated with RIP buffer containing magnetic beads conjugated with anti‐*c‐Myc*, anti‐EZH2 and anti‐DNMT1 or with a negative control IgG. Immunoprecipitation separates RNA‐binding proteins and their bound RNA. Subsequently, the retrieved RNA was assayed and miR‐1929‐5p expression was determined by qRT‐PCR.

### 2.17 Co‐immunoprecipitation (Co‐IP) assay

Cells were lysed in pre‐cooled NP‐40 lysis buffer (Solarbio) which was containing 1% PMSF. Cell lysates were incubated with indicated antibody for 2 h followed by incubation with Protein‐G beads for 2 h at 4 °C. After washing for three times, the proteins were separated by SDS/PAGE and proceeded for western blotting.

### 2.18 Intraparenchymal injections

The mice were anesthetized by inhaled isoflurane and then placed on a heating pad to maintain a body temperature of 37 °C. The abdomen was shaved, and a midline incision was made to expose and isolate the left renal. miR‐1929‐5p overexpressing adeno‐associated virus 9 (AAV9) vector solution was infused into the renal. After removing the needle, the injection site was pressed with a cotton swab to hemostasis. The surgical site was then sutured and the mice were returned to their cage, for use in further experiments 2 weeks later. The experimental protocol was approved by the Institutional Animal Care and Use Committee at the Ningxia medical university Laboratory Animal Center (ethics approval number is NYDWZX‐2018‐083).

### 2.19 RNA‐sequencing (RNA‐seq) analysis

Total RNA, including small RNA, was extracted from podocytes using TRIzol reagent (Invitrogen). RNA quality and quantity were analyzed using Qubit and Nanodrop, respectively. A small RNA library was prepared using Small RNA Sample Pre‐Kit (Ipswich, MA, USA) following manufacturer’s recommendations. Briefly, using the special structure of 3′ and 5′ ends of small RNA (with complete phosphate group at the 5′ end and hydroxyl group at the 3′ end), starting with total RNA for samples, the two ends of small RNA were directly connected and cDNA was synthesized by reverse transcription. The amplified and purified PCR products were sequenced on an illumina HiSeq™2500/MiSeq at Novogene Sciences (Beijing, China). After sequencing, the clean data (clean reads) were obtained by removing the low‐quality reads from raw data.

### 2.20 Statistical analysis

All data are expressed as mean ± SD. The results analysis used graph pad prism 5.0 software (Graph Pad software, San Diego, CA, USA). One‐way ANOVA, Student–Newman–Keuls test (comparisons between multiple groups) or unpaired Student’s *t*‐test (between two groups) was used as appropriate. A *P*‐value <0.05 was considered significant.

## 3 Results

### 3.1 Hcy facilitates podocyte apoptosis and glomerular damage in *cbs^+/−^* mice

**Fig. 1:**
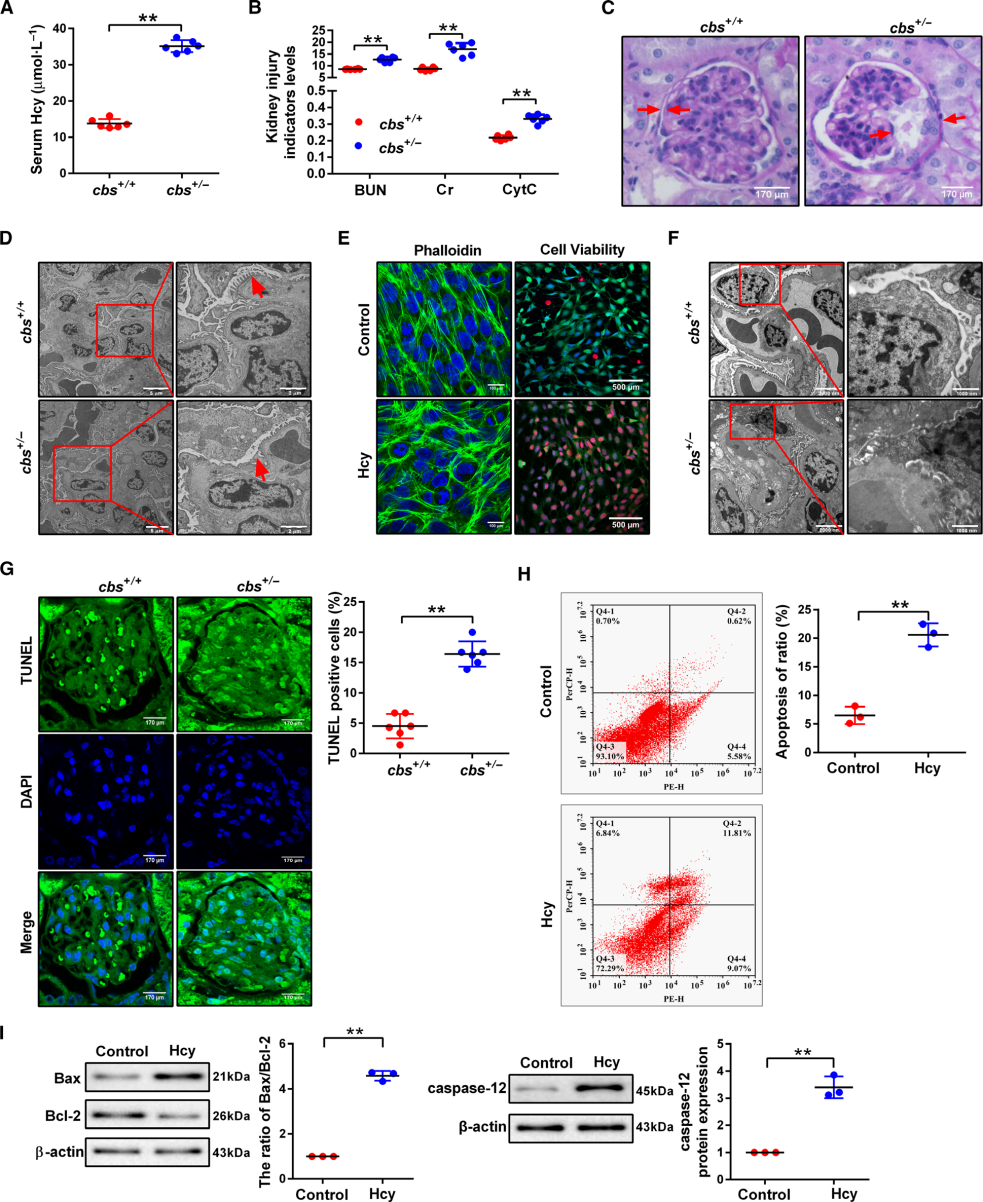
Hcy‐induced podocyte apoptosis and glomerular injury. (A,B) The levels of serum Hcy, blood urea nitrogen (BUN), creatinine (Cr) and cytochrome‐*c* (CytC) in the *cbs^+/+^* and *cbs^+/−^* mice were measured by automatic biochemical analyzer (*n* = 6). (C) Glomerular structural change of kidney sections using PAS staining in the *cbs^+/+^* and *cbs^+/−^* mice. Scale bars: 170 μm. (D) TEM of glomeruli. Scale bars: 5 μm, 2 μm. (E) Representative immunofluorescence images of viable (green), dead (red) and nuclei (blue) in the podocytes treated with Hcy (Scale bar, 500 μm) and photomicrographs of stress fibers by phalloidin‐iFluor™ 488 conjugate staining. Scale bar: 100 μm). (F) Apoptotic podocytes in the glomerulus were assessed by TEM in *cbs^+/+^* and *cbs^+/−^* mice. Scale bar, 2000 nm, 1000 nm. (G) Apoptotic podocytes in the glomerulus of *cbs^+/+^* and *cbs^+/−^* mice were assessed by TUNEL staining (*n* = 6). Scale bars: 170 μm. (H) Apoptosis rate of podocytes was measured by flow cytometry after podocytes were treated with Hcy (*n* = 3). (I) Representative western blot and quantification results of Bax, Bcl‐2 and caspase‐12 in the podocytes treated with Hcy (*n* = 3). **P < 0.01.

To explore the role of Hcy in glomerular damage, mice were first fed 2.0% methionine to induce HHcy after 8 weeks. The serum Hcy levels were significantly increased in the *cbs^+/−^* mice, indicating the establishment of the HHcy mice model (Fig. 1A). Increased levels of blood urea nitrogen (BUN), creatinine (Cr) and cytochrome‐*c* (CytC) were found in *cbs^+/−^* mice (Fig. 1B). The result of periodic acid‐Schiff (PAS) staining indicated normal glomerular and tubular structures in *cbs^+/+^* mice, whereas a typical pathological change showing glomerular sclerotic damage such as glomerular capillary collapse and mesangial expansion was observed in the *cbs^+/−^* mice (Fig. 1C). The images of transmission electron microscopy (TEM) showed thickened ultrastructure of glomerular basement membrane, effacement and focal fusion of the podocyte foot process in *cbs^+/−^* mice (Fig. 1D). Moreover, Hcy prominently promoted actin fiber disruption and reduced cell viability in podocytes (Fig. 1E). These results suggested that Hcy can induce glomerular podocyte injury. To determine the correlation between HHcy and glomerular dysfunction, the impact and molecular mechanisms were initially probed in the glomerular podocytes. Interestingly, the TEM images showed the occurrence of typical apoptotic morphology in the podocyte isolated from *cbs^+/−^* mouse glomeruli with shrunken nucleus and condensed chromatin (Fig. 1F). TUNEL staining assay indicated reduced TUNEL‐positive kidney parenchyma cells in the *cbs^+/+^* group (Fig. 1G). Flow cytometry analysis further confirmed the increase in podocyte apoptosis in the Hcy group (Fig. 1H). Moreover, the ratio of Bax/Bcl‐2 and caspase‐12 expression were significantly elevated in the Hcy group (Fig. 1I). These results indicated that podocyte apoptosis may be the main reason for Hcy‐induced glomerular dysfunction.

### 3.2 miR‐1929‐5p targeting Bcl‐2 leading podocyte apoptosis was a major mechanism in the glomerular dysfunction induced by Hcy

**Fig. 2:**
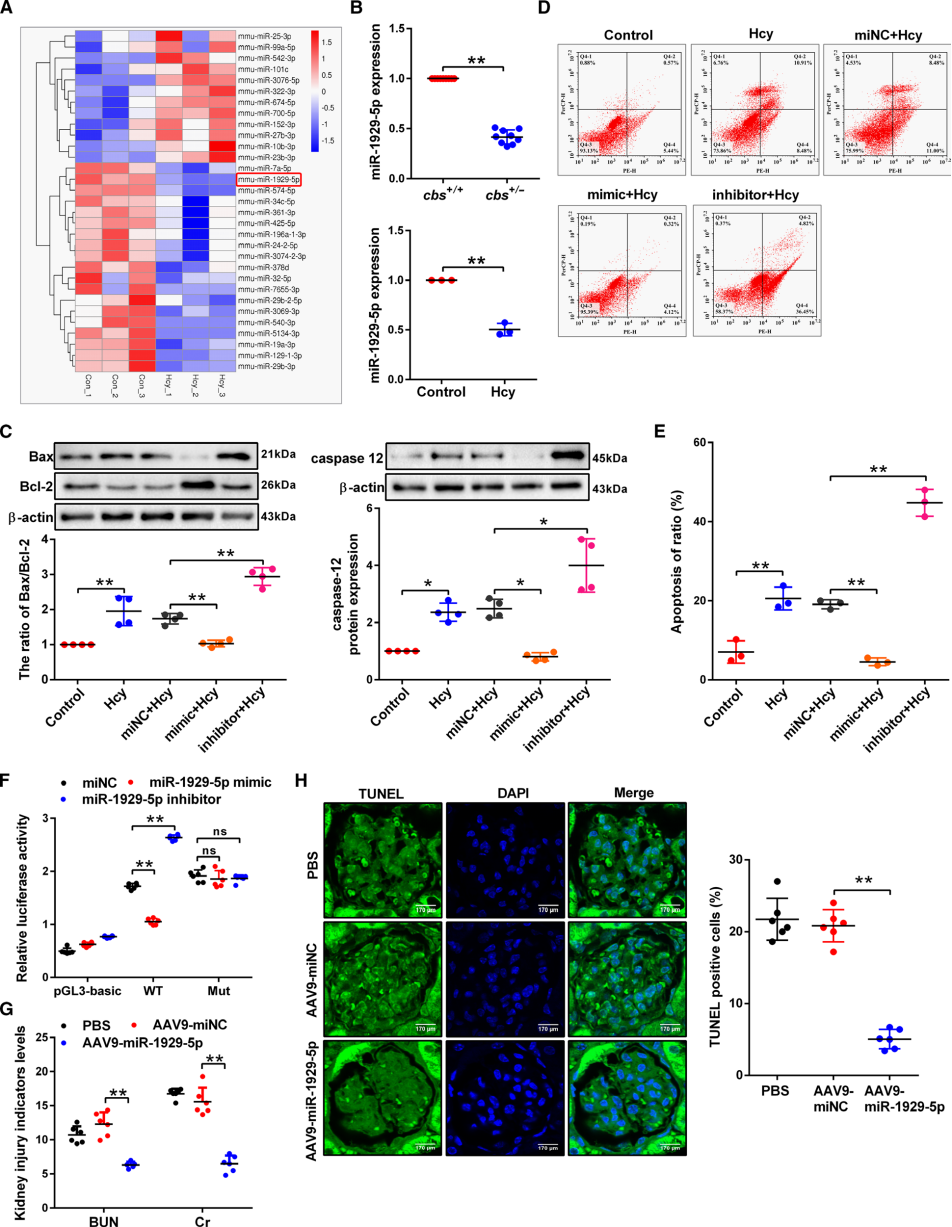
MicroRNA‐1929‐5p inhibits Hcy‐induced podocyte apoptosis. (A) Clustering of hierarchical cluster analysis of differentially expressed miRNA in the Control and Hcy group. Red indicates upregulated miRNA, and blue downregulated miRNA. (B) The level of miR‐1929‐5p in the glomeruli (*n* = 9) and Hcy‐treated podocytes (*n* = 3) was analyzed by qRT‐PCR. (C–E) Quantitative analysis of Bax, Bcl‐2 and caspase‐12 protein levels (*n* = 4) and analysis of apoptosis rate of podocytes (*n* = 3) after transfection with miNC, miR‐1929‐5p mimic or inhibitor and treatment with Hcy, respectively. (F) The reporter constructs containing 3′‐UTR regions of the wild type (WT) and mutant type (Mut) Bcl‐2 were co‐transfected with miR‐1929‐5p mimic or inhibitor. The ratio of firefly and renilla luciferase activities represents the relative luciferase activities (*n* = 6). (G,H) The levels of BUN, Cr and the number of apoptotic podocytes in glomerulus. Scale bars: 170 μm after AAV9‐miR‐1929‐5p was delivered into kidney of *cbs^+/−^* mice by intraparenchymal injection (*n* = 6). *P < 0.05, **P < 0.01.

Then, the RNA‐seq indicated that 31 miRNA out of 790 miRNA were differentially expressed in the Hcy group versus the Control group (Figs 2A and S1A,B). The Gene Ontology (GO) and KEGG pathway analysis further found that the correlated genes were related to metabolism and apoptosis (Fig. S1C). It was found that miR‐1929‐5p was downregulated both in the glomeruli and Hcy‐treated podocytes (Fig. 2B). We then transfected miNC, miR‐1929‐5p mimic or miR‐1929‐5p inhibitor into podocytes (Fig. S1D,E). It was found that miR‐1929‐5p mimic decreased the ratio of Bax/Bcl‐2, caspase‐12 levels and apoptosis rate in Hcy‐treated podocytes, and this can be reversed by the miR‐1929‐5p inhibitor (Fig. 2C–E), indicating that miR‐1929‐5p can protect podocytes from apoptosis. To determine the mechanism of miR‐1929‐5p in the podocyte apoptosis induced by Hcy, miR‐1929‐5p and its target gene Bcl‐2 were predicted by TargetScan, which was confirmed by luciferase assay (Figs 2F and S1F). Furthermore, by delivering the recombinant adeno‐associated virus serotype 9 (AAV9) vectors harboring miR‐1929‐5p *in vivo*, we found a decreased level of BUN, Cr and apoptosis rate in AAV9‐miR‐1929‐5p mice (Fig. 2G,H). These results indicated that inhibition of miR‐1929‐5p expression is associated with Hcy‐induced podocyte apoptosis through the Bcl‐2 signal pathway.

### 3.3 DNA hypermethylation of miR‐1929‐5p promoter induced by Hcy contributes to podocyte apoptosis in the kidney

**Fig. 3:**
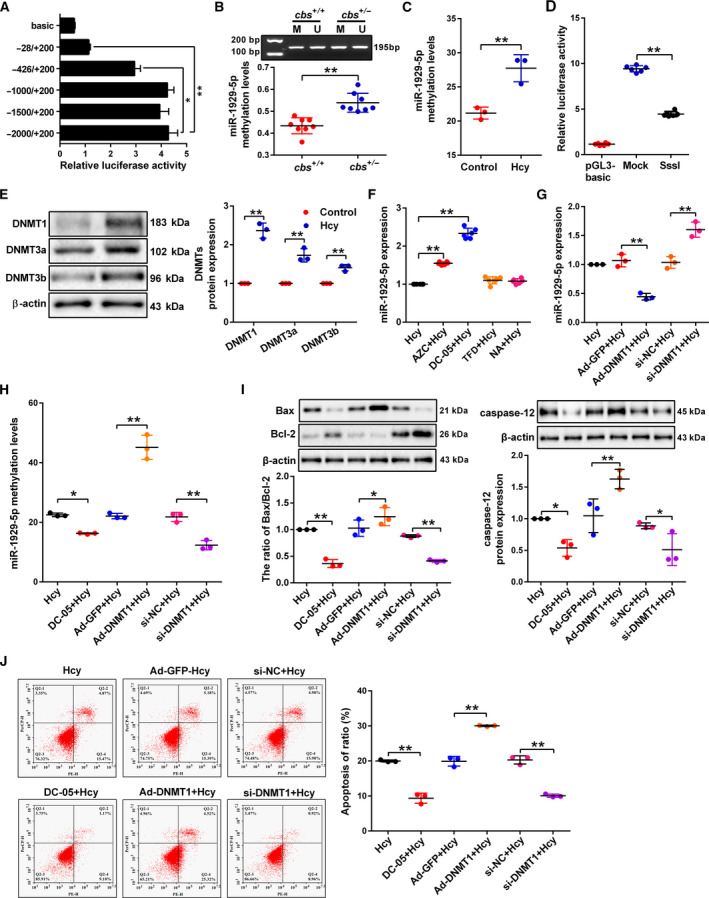
Hcy promotes miR‐1929‐5p hypermethylation by upregulating DNMT1 expression. (A) The promoter activity of miR‐1929‐5p by dual‐luciferase reporter assay. Various regions of the miR‐1929‐5p promoter (−2000/+200, −1500/+200, −1000/+200, −426/+200, −28/+200) were co‐transfected into HEK293 cells with renilla luciferase vector, respectively (*n* = 9). (B) DNA methylation of miR‐1929‐5p promoter region in the glomeruli from *cbs^+/+^* and *cbs^+/−^* mice was analyzed by nMS‐PCR (*n* = 8). The reactions for unmethylated and methylated DNA are denoted by U and M, respectively. (C) MassARRAY analysis of DNA methylation in the miR‐1929‐5p promoter region in podocytes treated with Hcy (*n* = 3). (D) MicroRNA‐1929‐5p promoter was methylated by M.SssI, and the transcriptional activity of miR‐1929‐5p promoter was detected by luciferase reporter assay (*n* = 6). (E) The expression of DNMT1, DNMT3a and DNMT3b was detected by western blot in the podocytes treated with Hcy (*n* = 3). (F) The level of miR‐1929‐5p in the podocytes was detected after treatment with Hcy together with AZC, DC‐05, theaflavin 3, 3′‐digallate or Nanaomycin A (DNMT, DNMT1, DNMT3a and DNMT3b specific inhibitors, respectively) (*n* = 6). (G,H) MicroRNA‐1929‐5p expression and DNA methylation levels in the podocytes were detected after transfected with si‐DNMT1 or Ad‐DNMT1 and treated with Hcy (*n* = 3). (I, J) The protein levels of Bax, Bcl‐2 and caspase‐12 (*n* = 3) and the apoptosis rate of podocytes (*n* = 3) were examined after treatment with Hcy together with the treatment of DC‐05 or transfection with si‐DNMT1 or Ad‐DNMT1. *P < 0.05, **P < 0.01.

Since DNA methylation plays an important role in the regulation of gene expression, we then investigated the DNA methylation level of miR‐1929‐5p promoter in Hcy‐treated podocytes. Luciferase assay indicated that the region between −426 and +200 of miR‐1929‐5p promoter is critical for miR‐1929‐5p expression (Fig. 3A). nMS‐PCR assay showed that the DNA methylation level of miR‐1929‐5p promoter is increased in the glomeruli of *cbs^+/−^* mice (Fig. 3B). Meanwhile, MassARRAY showed that the average methylation levels of these CpG sites increased after treatment with Hcy (Fig. 3C). We found that the promoter activity of miR‐1929‐5p promoter was significantly decreased after SssI DNA methylase (M.SssI) treatment (Fig. 3D). The DNA methyltransferase (DNMT) family, including DNMT1, DNMT3a and DNMT3b, are the enzymes responsible for regulating and maintaining DNA methylation levels. The result of western blot assay indicated that DNMT1, DNMT3a and DNMT3b expression was conspicuously increased (Fig. 3E). However, only 5‐azacytidine (AZC) and DC‐05 (DNMT1 inhibitor) treatment increased the expression of miR‐1929‐5p, suggesting that DNMT1 is the major regulator of the methylation of miR‐1929‐5p promoter (Fig. 3F). Further evidence showed that knockdown of DNMT1 increased the expression of miR‐1929‐5p and decreased miR‐1929‐5p promoter methylation, whereas overexpression of DNMT1 caused the opposite result (Fig. 3G,H). Moreover, DC‐05 and knockdown of DNMT1 significantly inhibited podocyte apoptosis, whereas overexpression of DNMT1 promoted podocyte apoptosis (Fig. 3I,J). These results demonstrated that the inhibition of miR‐1929‐5p expression by Hcy is due to the hypermethylation of DNMT1 on the miR‐1929‐5p promoter.

### 3.4 H3K27me3 in the promoter of miR‐1929‐5p by EZH2 contributes to Hcy‐induced miR‐1929‐5p downregulation and podocyte apoptosis

**Fig. 4:**
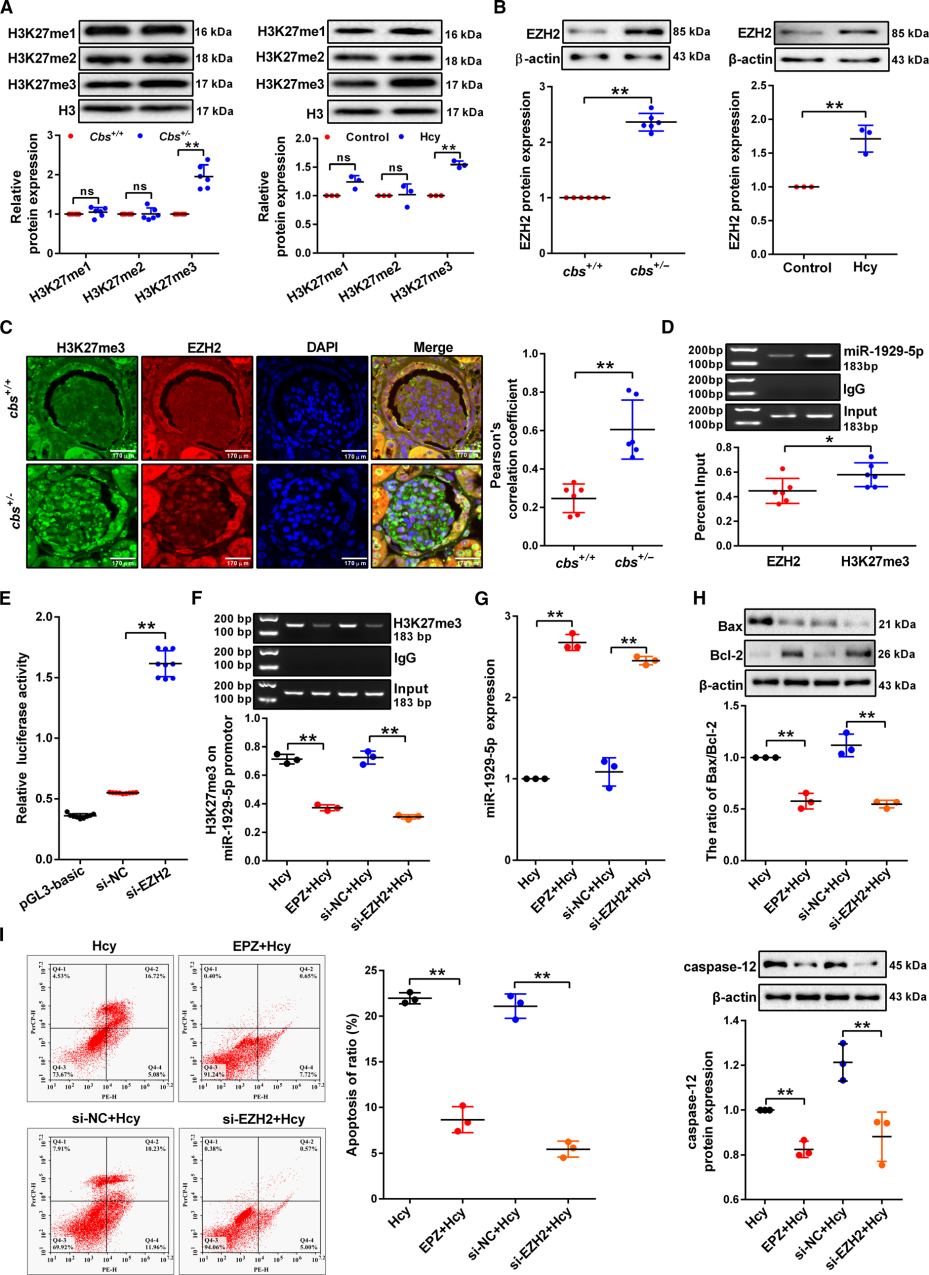
Status of H3K27me3 on the miR‐1929‐5p promoter by EZH2 contributed to podocyte apoptosis induced by Hcy. (A) Global levels of H3K27me1, 2, 3 in the glomeruli (*n* = 6) or podocytes (*n* = 3) treated with Hcy were detected by western blot. (B) EZH2 expression was detected by western blot in the glomeruli (*n* = 6) and podocytes (*n* = 3) treated with Hcy. (C) Co‐localization of EZH2 and H3K27me3 in the glomeruli from *cbs^+/+^* and *cbs^+/−^* mice by immunofluorescence staining with anti‐H3K27me3 (green), anti‐EZH2 (red) antibodies, and DAPI (blue) (*n* = 6). Scale bars: 170 μm. (D) The occupancy of EZH2 and H3K27me3 on miR‐1929‐5p promoter in the podocytes detected by ChIP analysis (*n* = 6). (E) MicroRNA‐1929‐5p promoter activity was examined by miR‐1929‐5p promoter‐driven luciferase reporter system after knockdown of EZH2 (*n* = 9). (F) ChIP analysis of H3K27me3 occupancy on miR‐1929‐5p promoter in the podocytes transfected with si‐EZH2 or treated with EZH2 specific inhibitor (EPZ005687, EPZ) and stimulation with Hcy (*n* = 3). (G) Expression of miR‐1929‐5p was examined in the podocytes after knockdown of EZH2 or treatment with EPZ (*n* = 3). (H,I) Protein levels of Bax, Bcl‐2 and caspase‐12 and apoptosis rate of podocytes (*n* = 3) were determined after treatment with Hcy together with knockdown of EZH2 expression or treatment with EPZ. *P < 0.05, **P < 0.01.

We examined whether histone methylation contributed to the decreased expression of miR‐1929‐5p caused by Hcy. Biological information analysis found that there is a marker of H3K27me3 in the promoter regions of miR‐1929‐5p (Fig. S2A). In the following experiment, we found that the levels of H3K27me3 were significantly increased in the glomerular isolated from *cbs^+/−^* mice and podocytes treated with Hcy (Fig. 4A). The further result of GO‐biological process indicated that EZH2 can strongly catalyze H3K27me3 (Fig. S2B). Meanwhile, western blot showed that Hcy treatment caused significant increase of EZH2 protein (Fig. 4B). Co‐immunofluorescence staining of glomerular podocytes showed the coexistence of EZH2 and H3K27me3 with increased level in the glomerular podocytes isolated from *cbs^+/−^* mice (Fig. 4C). ChIP assay also showed that both EZH2 and H3K27me3 generally occupied the miR‐1929‐5p promoter (Fig. 4D). In addition, we found that knockdown EZH2 increased the promoter activity of miR‐1929‐5p by luciferase assay (Fig. 4E). Using the ChIP assay, we also found that EZH2 inhibition markedly decreased the occupancy of H3K27me3 on the promoter of miR‐1929‐5p (Fig. 4F). Accordingly, the inhibition of EZH2 upregulated the expression level of miR‐1929‐5p (Fig. 4G), suggesting that EZH2 directly regulates miR‐1929‐5p expression. Furthermore, the ratio of Bax/Bcl‐2 level, caspase‐12 expression level and the apoptosis rate in the podocytes were obviously decreased after EZH2 inhibition (Fig. 4H,I). Taken together, these data suggest that EZH2 negatively regulates miR‐1929‐5p expression via H3K27me3 on the promoter of miR‐1929‐5p, leading to enhanced podocyte apoptosis induced by Hcy.

### 3.5 Hcy inhibits miR‐1929‐5p expression via the cooperation of DNMT1 and EZH2 in the podocytes

**Fig. 5:**
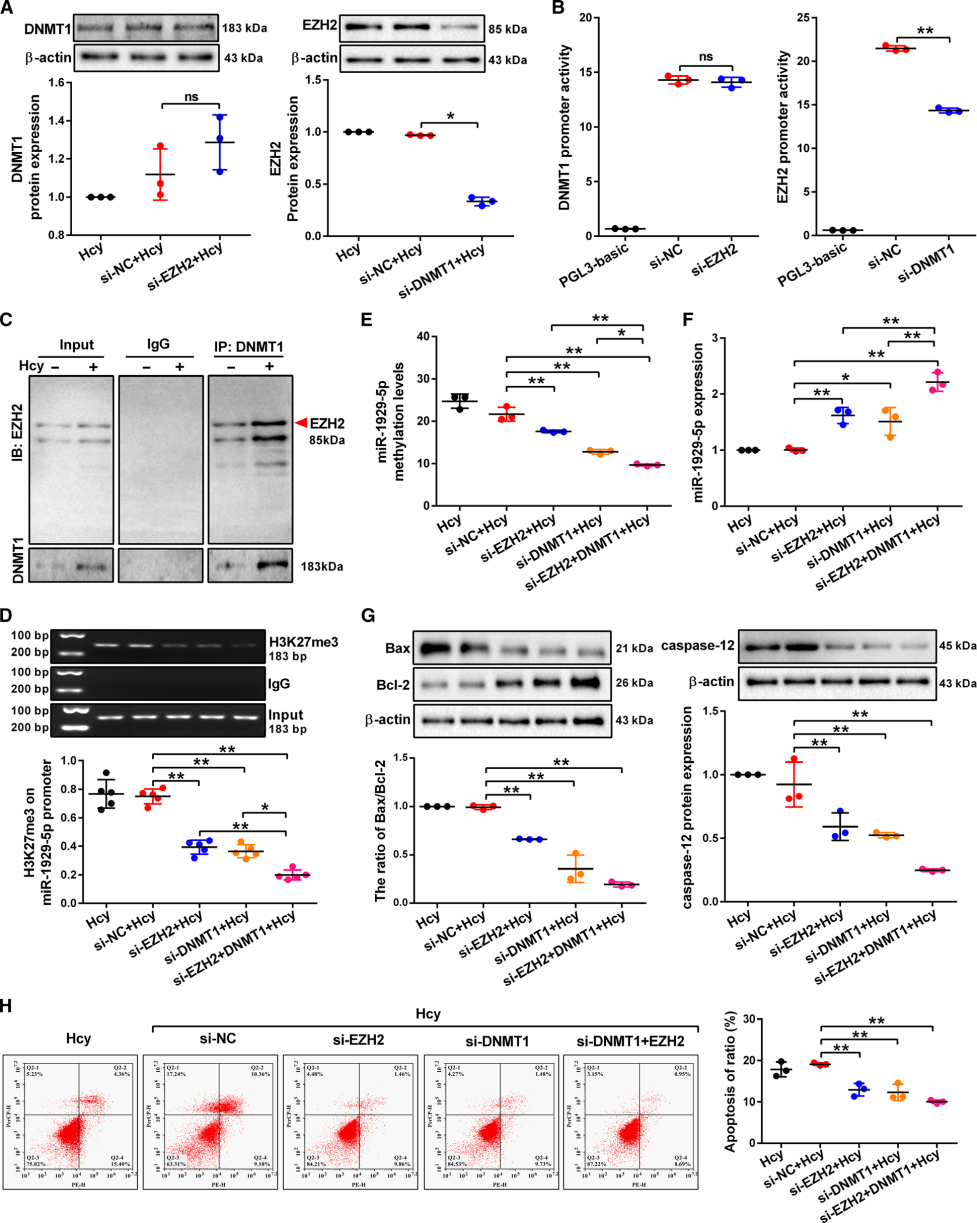
DNMT1 cooperates with EZH2 to regulate miR‐1929‐5p expression in Hcy‐treated podocytes. (A) DNMT1 and EZH2 protein expression was detected by western blot after podocytes were transfected with control siRNA (si‐NC), si‐DNMT1 or si‐EZH2 and exposed to Hcy (*n* = 3). (B) The promoter activity of EZH2 and DNMT1 was measured by dual luciferase assay after cells transfected with si‐NC, si‐DNMT1 or si‐EZH2 (*n* = 3). (C) The binding of DNMT1 with EZH2 was examined by Co‐IP in podocytes treated with Hcy. (D) The levels of H3K27me3 on miR‐1929‐5p promoter were detected by ChIP analysis when DNMT1 or EZH2 expression were knocked down in the podocytes (*n* = 5). (E) The average miR‐1929‐5p methylation levels in promoter region were analyzed by MassARRAY analysis (*n* = 3). (F) The miR‐1929‐5p expression in the podocytes was detected by qRT‐PCR (*n* = 3). (G) Representative western blot showing the protein levels of Bax, Bcl‐2 and caspase‐12 after podocytes were transfected with si‐NC, si‐DNMT1 or si‐EZH2 and exposed to Hcy (*n* = 3). (H) Podocytes were transfected with si‐NC, si‐DNMT1 or si‐EZH2 and the apoptosis rate of podocytes was measured by flow cytometry analysis (*n* = 3). *P < 0.05, **P < 0.01.

Since DNMT1 and EZH2 are involved in Hcy‐induced miR‐1929‐5p downregulation, we studied the potential interaction between DNMT1 and EZH2. Silencing of DNMT1 expression reversed the effect of Hcy on EZH2 protein levels, whereas there was no noticeable effect on DNMT1 protein level when EZH2 expression was knocked down (Fig. 5A). Thereafter, luciferase assay showed that knockdown of DNMT1 decreased EZH2 promoter activity, whereas knockdown of EZH2 did not affect activity of DNMT1 promoter activity (Fig. 5B). In addition, Hcy treatment could increase the interaction between DNMT1 and EZH2 in the podocytes (Fig. 5C). We further found that the H3K27me3 level on the miR‐1929‐5p promoter was decreased after knockdown of DNMT1 or EZH2 expression (Fig. 5D). The level of DNA methylation showed a parallel alteration in the podocytes when DNMT1 and EZH2 expression was knocked down, and the miR‐1929‐5p expression was notably enhanced (Fig. 5E,F). Similar results were consistently obtained after DC‐05 and EPZ treatment (Fig. S3A–C). Moreover, silencing of DNMT1 and EZH2 expression decreased the ratio of Bax/Bcl‐2, caspase‐12 expression and podocyte apoptosis rate (Fig. 5G,H). These results suggested that DNMT1 not only regulates the expression of EZH2 but also interacts with EZH2, thereby contributing to the apoptosis caused by Hcy in the podocytes.

### 3.6 *c‐Myc*‐mediated transcriptional repression of miR‐1929‐5p contributes to podocyte apoptosis induced by Hcy

**Fig. 6:**
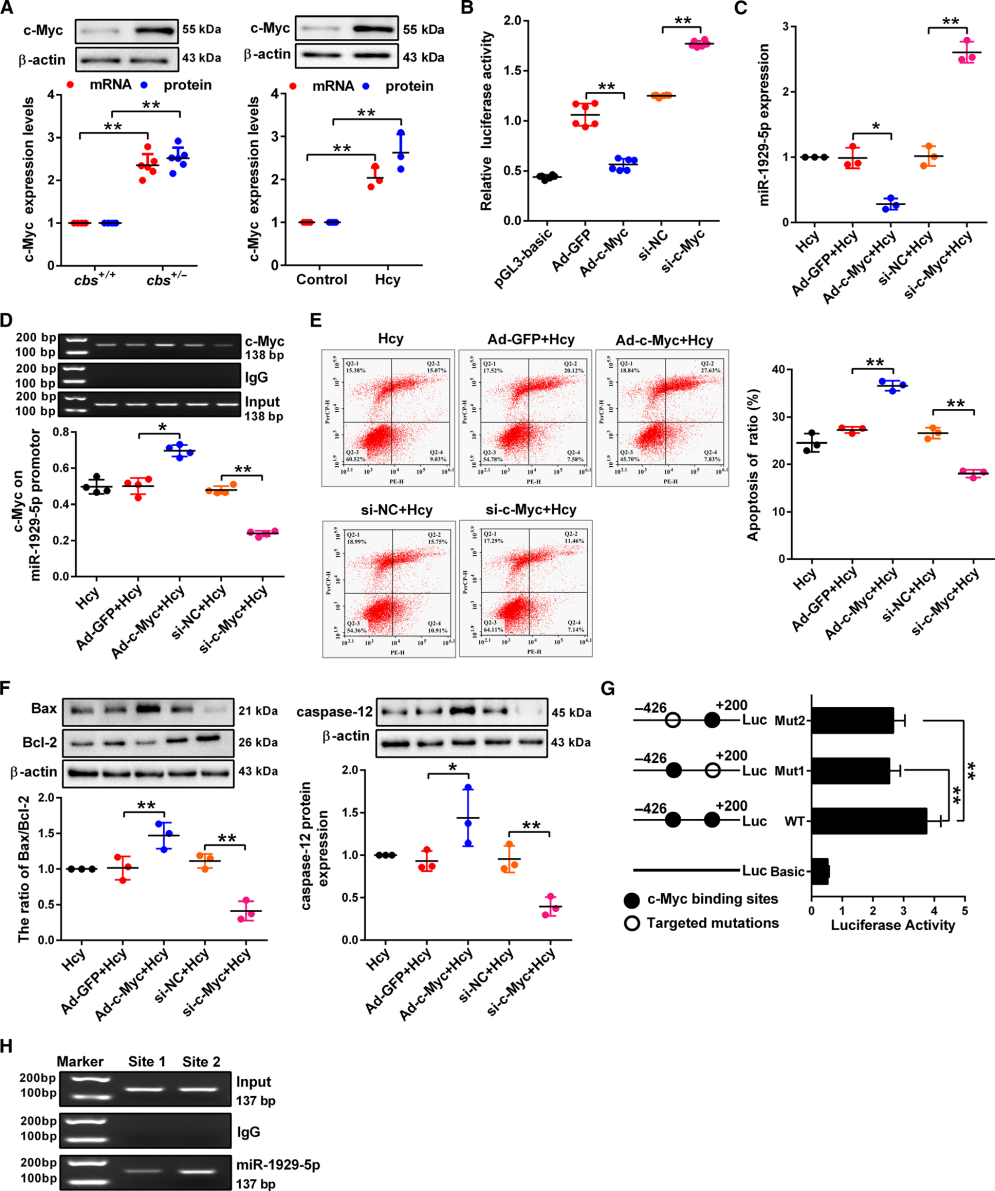
The c‐Myc‐mediated transcriptional repression of miR‐1929‐5p contributes to podocyte apoptosis induced by Hcy. (A) The mRNA and protein expression of *c‐Myc* was measured by qRT‐PCR and western blot in the glomeruli (*n* = 6) and podocytes treated with Hcy (*n* = 3). (B) The miR‐1929‐5p promoter activities were examined by luciferase reporter assay after transfection with Ad‐*c‐Myc* or si‐*c‐Myc* (*n* = 6). (C) The expression of miR‐1929‐5p in the podocytes infected with Ad‐*c‐Myc* and si‐*c‐Myc* was examined by qRT‐PCR (*n* = 3). (D) ChIP assay showed recruitment of *c‐Myc* to the promoter region of miR‐1929‐5p (*n* = 4). (E) The apoptosis rate of podocytes infected with Ad‐*c‐Myc* and si‐*c‐Myc* upon Hcy treatment was analyzed by flow cytometry analysis (*n* = 3). (F) Bax, Bcl‐2 and caspase‐12 protein expression in the podocytes after infection with Ad‐*c‐Myc* and si‐*c‐Myc* following Hcy treatment (*n* = 3). (G) The luciferase activities of the miR‐1929‐5p promoter with WT or the Mut binding site of *c‐Myc* were determined using luciferase reporter assays (*n* = 9). (H) ChIP assays showing the *c‐Myc*‐binding sites 1 and 2 of miR‐1929‐5p promoter in the podocytes. *P < 0.05, **P < 0.01.

Next, we found that *c‐Myc* expression is increased significantly in glomeruli from *cbs^+/−^* mice and podocytes stimulated with Hcy (Fig. 6A). In addition, overexpression of *c‐Myc* decreased miR‐1929‐5p promoter activity, while knockdown *c‐Myc* resulted in the opposite result (Fig. 6B). Correspondingly, after podocytes were transfected with Ad‐*c‐Myc* and si‐*c‐Myc* (Fig. S4A, B), the expression of miR‐1929‐5p was strikingly decreased in the podocytes transfected with Ad‐*c‐Myc*, supporting the role of *c‐Myc* in suppression of miR‐1929‐5p transcription (Fig. 6C,D). As an upstream regulator of miR‐1929‐5p/Bax/Bcl‐2 axis, *c‐Myc* knockdown markedly reduced Hcy‐induced podocyte apoptosis, whereas the opposite result was obtained when *c‐Myc* was overexpressed in the cells (Fig. 6E,F). Next, we found two putative binding sites of *c‐Myc* at the promoter of miR‐1929‐5p, which are close to the transcription start site (TSS) (Fig. S4C). The luciferase reporter assay showed a significant reduction of miR‐1929‐5p promoter activity when two putative binding sites were mutated (Fig. 6G). In addition, ChIP assay showed strong binding of *c‐Myc* to miR‐1929‐5p promoter at site 2 (Fig. 6H). These results indicated that *c‐Myc* transcriptionally represses miR‐1929‐5p by direct binding to the miR‐1929‐5p promoter region.

### 3.7 *c‐Myc* recruits EZH2 and DNMT1 binding to the miR‐1929‐5p promoter in the podocytes treated with Hcy

**Fig. 7:**
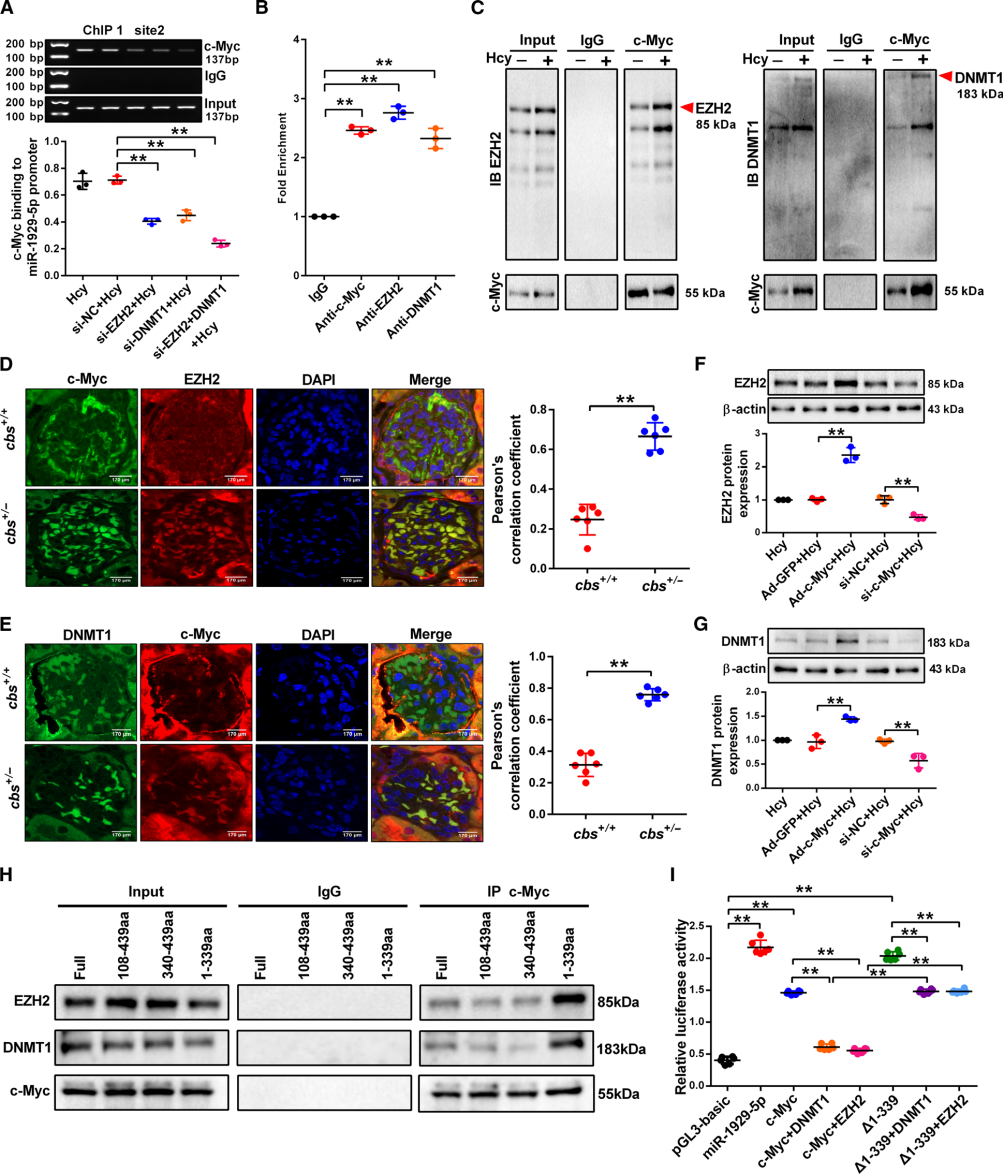
The*c‐Myc* recruits EZH2 and DNMT1 to bind to the miR‐1929‐5p promoter in the podocytes treated with Hcy. (A) Binding of *c‐Myc* on site 2 of miR‐1929‐5p promoter in the podocytes transfected with si‐DNMT1 and si‐EZH2 was detected by ChIP assay (*n* = 3). (B) RNA immunoprecipitation (RIP) with anti‐*c‐Myc*, anti‐EZH2 and anti‐DNMT1 followed by qRT‐PCR detection of miR‐1929‐5p RNA enrichment in immunoprecipitated complex compared with IgG (*n* = 3). (C) Co‐IP of interactions between *c‐Myc*, EZH2 and DNMT1 in the podocytes treated with Hcy. (D,E) Co‐localized EZH2 or DNMT1 with *c‐Myc* in the glomeruli were subjected to immunofluorescence staining with anti‐*c‐Myc*, anti‐EZH2, anti‐DNMT1 and DAPI (*n* = 6). Scale bars: 170 μm. (F,G) The protein expression of DNMT1 and EZH2 in the podocytes after transfection with Ad‐*c‐Myc* or si‐*c‐Myc* and exposure to Hcy (*n* = 3). (H) Mapping the interface of *c‐Myc* with EZH2 and DNMT1 by Co‐IP experiments co‐transfected with Myc‐tagged EZH2, Myc‐tagged DNMT1 and different FLAG‐tagged *c‐Myc* fragments. (I) Relative luciferase activity mediated by reporter constructs harboring the promoter of miR‐1929‐5p in HEK293 cells (*n* = 6). **P < 0.01.

We then performed experiments to determine whether *c‐Myc* cooperates with EZH2 and DNMT1 to regulate miR‐1929‐5p transcription during Hcy treatment. By ChIP assay, we found that knockdown of DNMT1 and EZH2 markedly decreased the binding ability of *c‐Myc* to the miR‐1929‐5p promoter at site 2, suggesting that DNA and histone methylation promote *c‐Myc* binding to the site 2 at miR‐1929‐5p promoter (Fig. 7A). In addition, RIP assay showed dramatic enrichment of miR‐1929‐5p with the *c‐Myc*, EZH2 and DNMT1 in immunoprecipitants (Fig. 7B). Moreover, the co‐binding of EZH2 and DNMT1 with *c‐Myc* suggests that EZH2, DNMT1 and *c‐Myc* function as a complex in the podocytes (Fig. 7C). Of note, immunofluorescence analysis demonstrated that a high level of *c‐Myc* correlates with increased EZH2 and DNMT1 staining in the glomeruli of *cbs^+/−^* mice (Fig. 7D,E). Interestingly, overexpression of *c‐Myc* upregulated the levels of EZH2 and DNMT1, whereas silencing of *c‐Myc* decreased EZH2 and DNMT1 expression (Fig. 7F,G). Collectively, these results suggested that *c‐Myc* recruits EZH2 and DNMT1 to repress miR‐1929‐5p expression. To clarify the key domains required for the binding of *c‐Myc* protein with EZH2 and DNMT1, several vectors containing FLAG‐tagged *c‐Myc* functional domains including MBI, MBII and bHLH‐LZ (Fig. S5A,B) were constructed. The results indicated that both the MBI and MBII domains of *c‐Myc* are required for its interaction with EZH2 and DNMT1 (Fig. 7H). Combined overexpression of EZH2 or DNMT1 and *c‐Myc* markedly inhibited miR‐1929‐5p promoter activity, whereas the deletion of MBI and MBII domains (▵1‐339) greatly impaired their repression ability of miR‐1929‐5p cooperatively (Fig. 7I). These results indicated that the two domains of *c‐Myc* were responsible for the interaction with EZH2 and DNMT1. These data demonstrated that *c‐Myc* recruits EZH2 and DNMT1 to repress miR‐1929‐5p expression through histone and DNA methylation.

## 4 Discussion

Recent study indicated that CKD is a known risk factor for the development of RCC [20]. Hcy is a sulfur‐containing amino acid that is formed as the trans‐methylation product during the metabolic conversion of methionine to cysteine. It is well known that the accumulation of Hcy promotes apoptosis and causes podocyte injury, which is closely related to the progression of CKD. Consistent with previous reports, in this study we found that the promotion of Hcy to glomerular podocyte injury is mediated by podocyte apoptosis. However, the underlying molecular mechanism of Hcy‐induced podocyte injury is still unclear.

MicroRNA are short single‐stranded RNA that can form hybrids with a specific region of target mRNA, especially within their 3′‐UTR. There is much evidence that miRNA are involved in the regulation of cell growth, apoptosis, proliferation and differentiation through targeted regulation of downstream gene expression [21]. Recent studies have also provided evidence that accumulating miRNA are related to the pathogenesis of kidney injury. For example, miR‐93 expression decreased HG‐treated podocytes of diabetic mice [22]. Conversely, miR‐29c is upregulated in diabetic mice and HG‐treated podocytes, where it contributes to Rho kinase activation and podocyte apoptosis [23]. By observing the miRNA expression profiles of Hcy‐treated podocytes, we found that the level of miR‐1929‐5p was significantly downregulated, implying a key role in podocyte injury. Recent studies indicated that miRNA play an important role in the regulation of renal function and our studies highlight a functional role of miR‐1929‐5p in HHcy‐induced kidney injury. We demonstrated that the podocyte apoptosis caused by Hcy was alleviated by miR‐1929‐5p overexpression. Recombinant adeno‐associated viral vector AAV9 harboring miR‐1929‐5p were delivered to the kidneys of *cbs^+/−^* mice and decreased BUN, Cr and apoptotic podocytes were found in AAV9‐miR‐1929‐5p mice. These data strongly indicated that miR‐1929‐5p as a central target molecule protects against podocyte injury induced by Hcy. Moreover, Bcl‐2 protein is considered an important anti‐apoptotic factor which prevents apoptosis without affecting cellular proliferation. In this study, we demonstrated that Bcl‐2 was a direct target of miR‐1929‐5p. Our results provide for the first time experimental evidence that HHcy downregulates miR‐1929‐5p in the podocytes, which may be an important pathogenic mechanism responsible for podocyte apoptosis leading to glomerular injury during HHcy.

As a precursor of *S*‐adenosylmethionine (SAM), a universal methyl donor, a high level of Hcy can lead to the increase in SAM level, which accordingly upregulates the activity of DNMTs using the substrate of DNMT [24, 25]. Our previous study showed that DNA methylation can contribute to the development of diseases such as cardiovascular and liver diseases caused by Hcy [15, 26]. In this study, we disclosed that Hcy treatment can cause the change of miR‐1929‐5p promoter from hypomethylating to hypermethylating podocytes, and the region between −426 and +200 of the miR‐1929‐5p promoter plays a key role in transcription regulation. Apart from DNA methylation, Hcy is also associated with histone methylation, which can alter the expression of remodeling genes. Our study showed that upregulation of EZH2 inhibits miR‐1929‐5p expression through elevating H3K27me3 at miR‐1929‐5p promoter in podocytes with Hcy treatment. It was reported that EZH2 may influence DNA methylation by direct interaction with DNMT [27]. In agreement with this point, we and others demonstrated the upstream role of DNMT1 on EZH2. Knockdown of EZH2 and DNMT1 can induce the increase of miR‐1929‐5p expression, which can be attributed to the suppression of DNA methylation and H3K27me3 on the miR‐1929‐5p promoter by Hcy. These results suggested that EZH2 and DNMT1 play a synergistic role in downregulating the expression of miR‐1929‐5p in podocytes. It is possible that DNA hypermethylation resulting from Hcy‐induced EZH2 upregulation can reinforce the epigenetic regulation of gene transcription. The detailed mechanisms involved in this interaction remain to be further elucidated.

Recent studies showed that *c‐Myc* can collaborate with epigenetic machinery to silence target genes [28]. For instance, *c‐Myc* recruits DNMT3b to the promoter region of RASSF1A, which causes DNA hypermethylation and reduces RASSF1A expression in lung cancer cells [29]. In addition, it was identified as a key regulator of EZH2 overexpression [30]. Until now, no study on the role of *c‐Myc* in the regulation of miR‐1929‐5p expression was reported. In this study, we demonstrated that DNA methylation and H3K27me3 modification contributed to the miR‐1929‐5p silencing mediated by *c‐Myc*. According to our knowledge, this is the first report about the regulation of *c‐Myc* on miR‐1929‐5p. In addition, we disclosed the marked enrichment of *c‐Myc*, DNMT1 and EZH2 on the miR‐1929‐5p promoter region in the Hcy‐treated podocytes, whereas *c‐Myc* could recruit the complex of DNMT1 and EZH2 to regulate miR‐1929‐5p expression by increasing DNA methylation and the H3K27me3 level of miR‐1929‐5p promoter. In addition, it is reported that direct binding of *c‐Myc* to the regulatory elements of EZH2 activates their transcription by recruiting chromatin modifier enzymes to their E‐box elements, leading to the increase of active RNA polymerase II on their promoters, further supporting our conclusions [31].

## 5 Conclusions

In summary, our data provide direct evidence that downregulation of miR‐1929‐5p facilitates podocyte apoptosis induced by Hcy. These results provide a mechanistic understanding of the mechanism whereby *c‐Myc* recruits EZH2 and DNMT1 to epigenetically suppressed miR‐1929‐5p expression through histone and DNA methylation during podocyte apoptosis induced by Hcy, suggesting a new pathogenic pathway contributing to glomerular injury associated with HHcy.

## Conflict of interest

The authors declare no conflict of interest.

## Author contributions

ZHP, JYD, XL and MSC designed experiments and wrote the manuscript. XL, DN, WYH, LGJ, XLB, WQQ, LK, JYZ, ZH, YAN and GYJ did the experiments and data analysis. All authors read and approved the final manuscript.

### Peer Review

The peer review history for this article is available at https://publons.com/publon/10.1002/1878‐0261.13032.

## Supporting information


**Fig. S1.** MicroRNA‐1929‐5p is the key molecule of Hcy in promoting podocyte apoptosis.
**Fig. S2.** EZH2 catalyzes H3K27me3 in the promoter region of miR‐1929‐5p.
**Fig. S3.** DNMT1 and EZH2 act synergistically to regulate miR‐1929‐5p expression in Hcy‐treated podocytes.
**Fig. S4.** *c‐Myc* expression in Hcy‐treated podocytes.
**Fig. S5.** Different domain structures of *c‐Myc*.
**Table S1.** Primer sequences for qRT‐PCR.
**Table S2.** Primer sequences for nMS‐PCR.

## Data Availability

The data that support the findings of this study are available from the corresponding author upon reasonable request.
