# Supplementary material for: Homocysteine induces podocyte apoptosis by regulating miR‐1929‐5p expression through c‐Myc, DNMT1 and EZH2
Source: Mol Oncol. 2021 Jul 19;15(11):3203–21. doi: 10.1002/1878-0261.13032 (PMC8564658; doi:10.1002/1878-0261.13032)
Supplement: Supplementary file 1 — Fig. S1. MicroRNA‐1929‐5p is the key molecule of Hcy in promoting podocyte apoptosis. Fig. S2. EZH2 catalyzes H3K27me3 in the promoter region of miR‐1929‐5p. Fig. S3. DNMT1 and EZH2 act synergistically to regulate miR‐1929‐5p expression in Hcy‐treated podocytes. Fig. S4. c‐Myc expression in Hcy‐treated podocytes. Fig. S5. Different domain structures of c‐Myc. Table S1. Primer sequences for qRT‐PCR. Table S2. Primer sequences for nMS‐PCR. [file MOL2-15-3203-s001.doc]

**SUPPLEMENTARY MATERIAL**

**1. Supplementary Figures**

**Figure S1. miR-1929-5p is the key molecule of Hcy in promoting podocytes apoptosis**


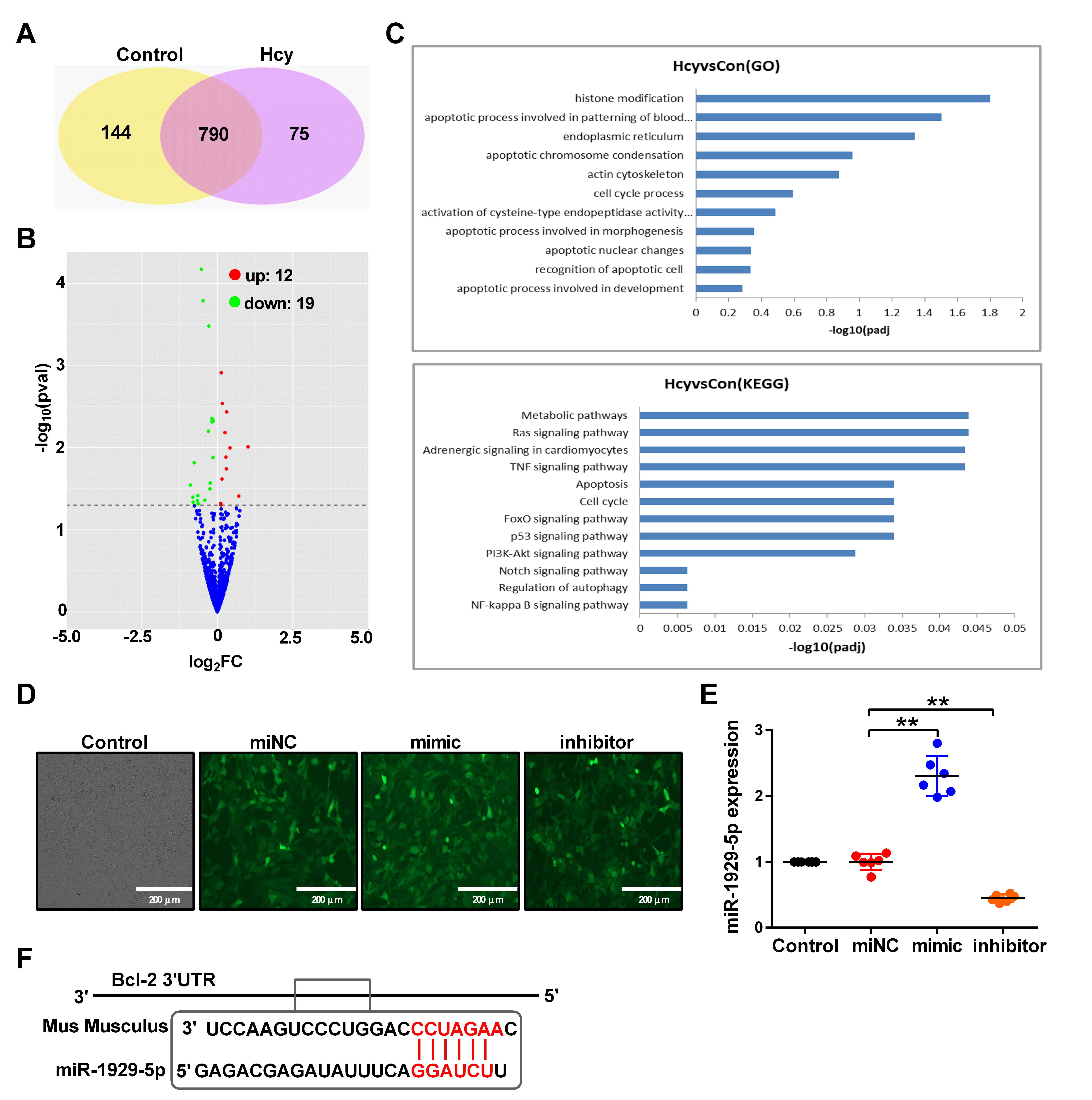


**Figure S1. miR-1929-5p is the key molecule of Hcy in promoting podocytes apoptosis. (A)** miRNA numbers of Control and Hcy group. **(B)** Volcano plot of differentially expressed miRNAs between Control and Hcy group. The volcano plot was constructed using fold-change values and *P*-values. The green and red points in the plot represent the differentially expressed miRNAs with statistical significance. **(C)** miRNAs co-expression genes assay using GO term and KEGG pathway enrichment. (**D)** Fluorescence intensity shows the transfection efficiency of transfected with miNC, miR-1929-5p mimic or miR-1929-5p inhibitor in the podocytes. (**E)** Quantitative assay of miR-1929-5p level in the podocytes after transfected with miNC, miR-1929-5p mimic or miR-1929-5p inhibitor (n=6). **(F)** Predicted miR-1929-5p target Bcl-2 3'-UTR. ^**^*P*<0.01.

**Figure S2. EZH2 catalyzes H3K27me3 in the promoter region of miR-1929-5p**


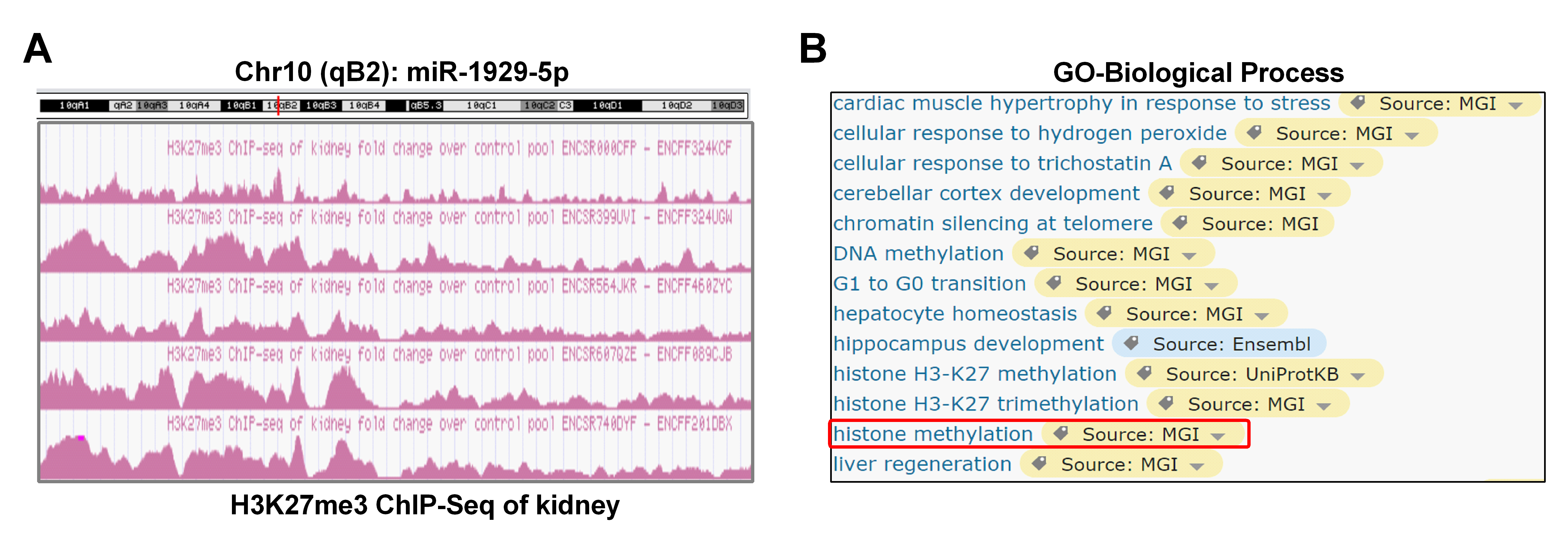


**Figure S2. EZH2 catalyzes H3K27me3 in the promoter region of miR-1929-5p. (A)** UCSC genome browser shows location of the miR-1929-5p gene at chromosome 10qB2. ChIP-Seq data generated from ENCODE shows that H3K27me3 marks are enriched in the promoter region of miR-1929-5p. **(B)** Bioinformatics analysis of EZH2 by Gene Ontology (GO)-biological process.

**Figure S3. DNMT1 and EZH2 synergistically to regulate miR-1929-5p expression in Hcy treated podocytes**


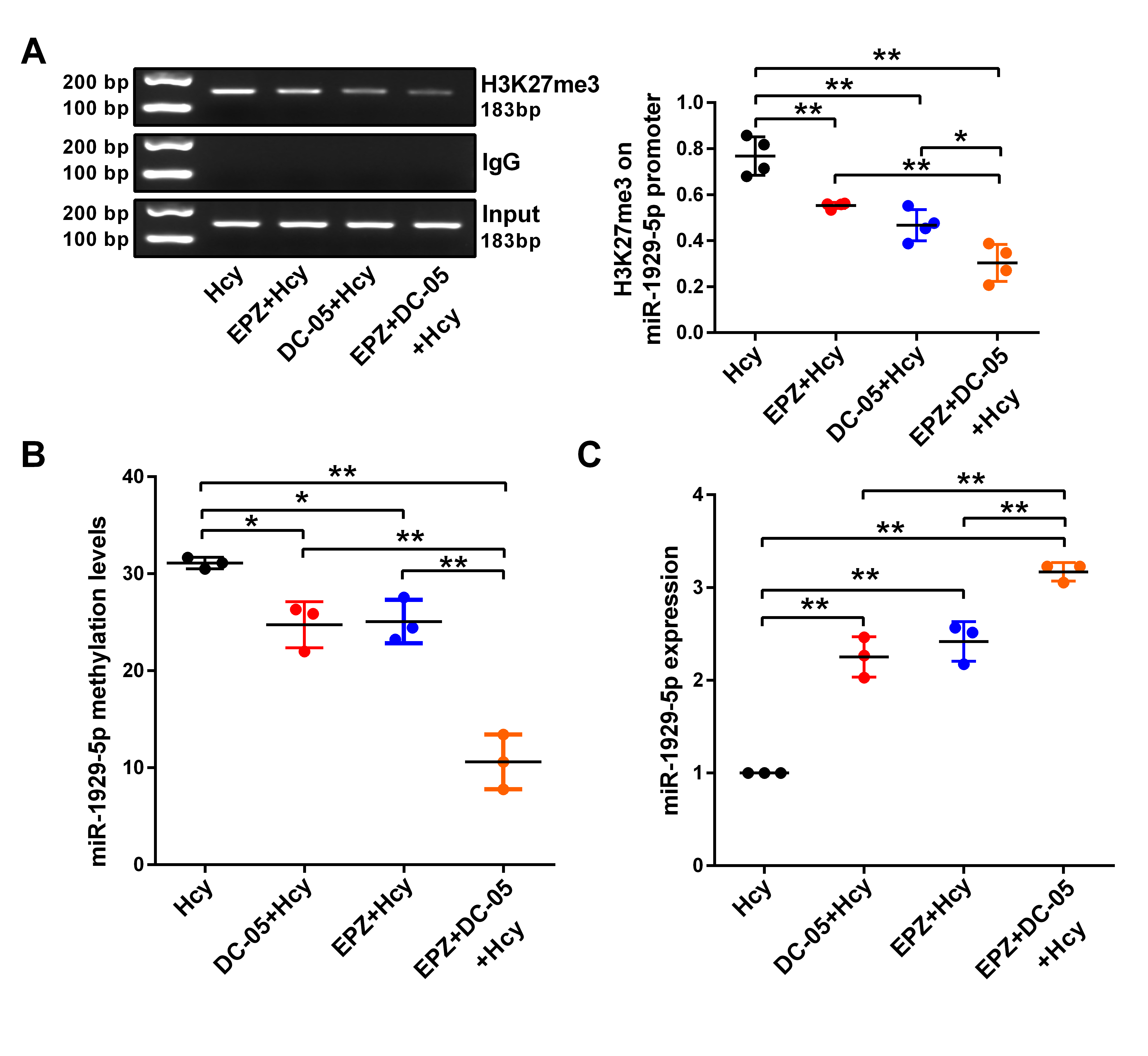


**Figure S3. DNMT1 and EZH2 synergistically to regulate miR-1929-5p expression in Hcy treated podocytes. (A)** The levels of H3K27me3 on miR-1929-5p promoter were detected by ChIP analysis after treatment with DC-05 and EPZ in presence of Hcy (n=4). (**B)** After podocytes were treated with DC-05 or EPZ and Hcy for 48 h, DNA methylation levels of miR-1929-5p promoter was detected by MassARRAY (n=3). (**C)** Impact of miR-1929-5p expression levels after podocytes treated with DC-05 or EPZ and Hcy for 48h (n=3). ^*^*P*<0.05, ^**^*P*<0.01.

**Figure S4. c-Myc expression in Hcy treated podocytes**


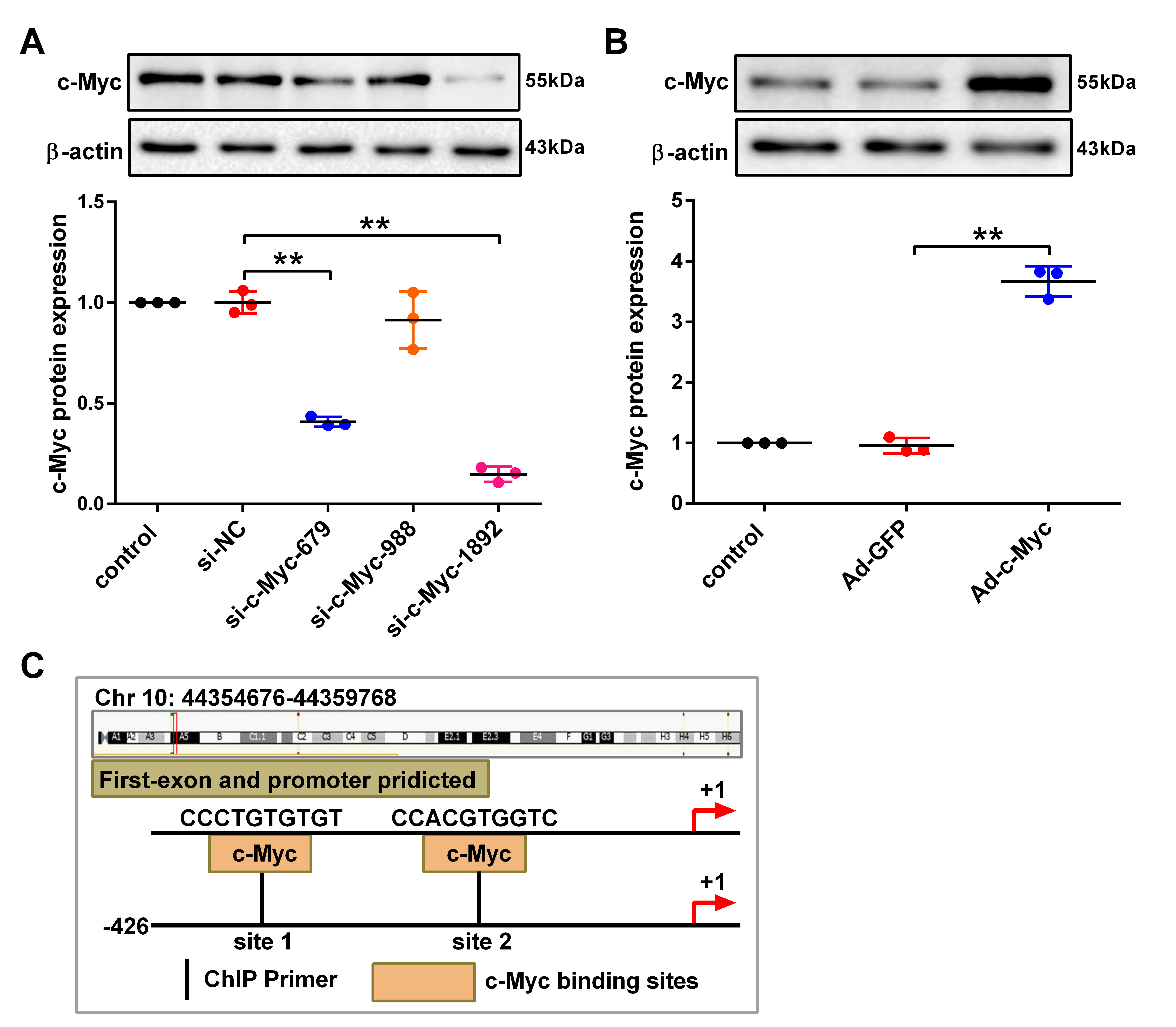


**Figure S4. c-Myc expression in Hcy treated podocytes. (A)** c-Myc protein levels were detected by western blot after podocytes were transfected with si-c-Myc (679, 988, 1892) (n=3). (**B)** c-Myc protein levels were detected by western blot after cells were transfected with Ad-c-Myc (n=3). **(C)** Diagram of the predicted two c-Myc binding sites in the miR-1929-5p promoter region, and ChIP primers were designed for these two binding sites. ^**^*P*<0.01.

**Figure S5. The different domain structure of c-Myc**


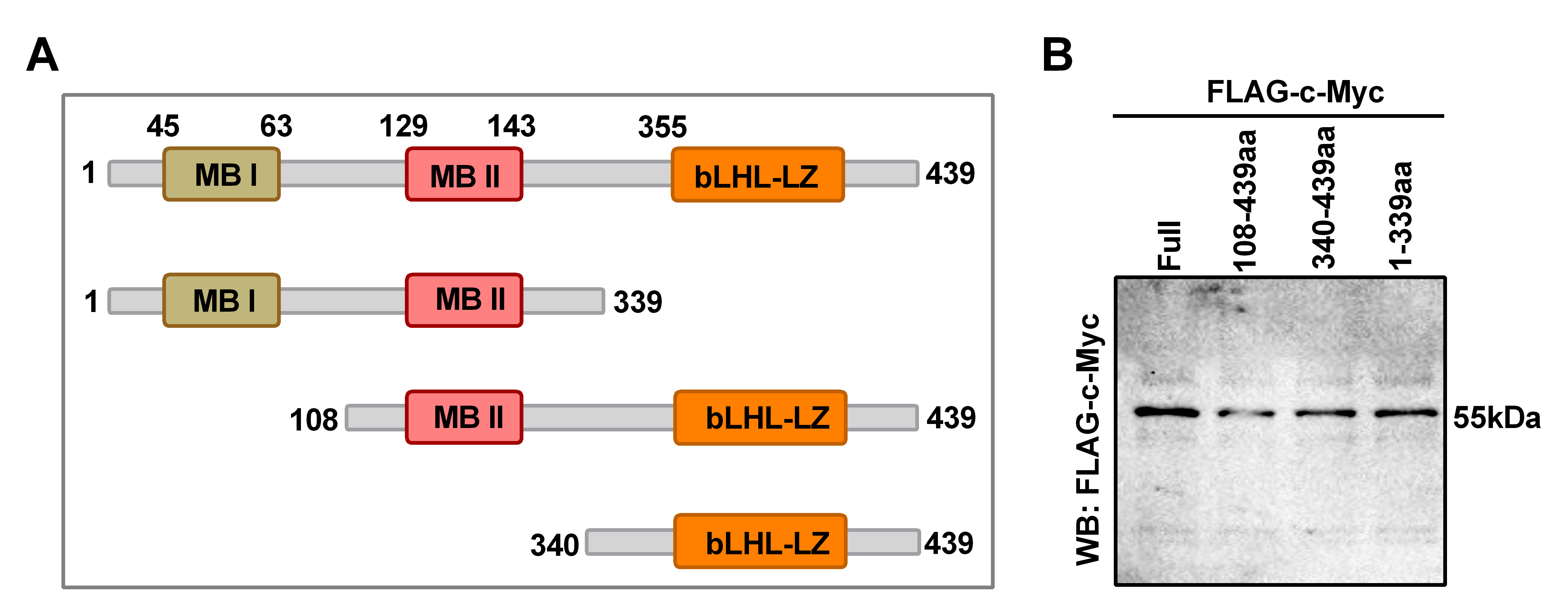


**Figure S5. The different domain structure of c-Myc. (A)** A pattern diagram of the different domain structure of FLAG-tagged c-Myc fragments. **(B)** FLAG-tagged c-Myc protein levels were detected by western blot after cells were transfected with different domain structure of FLAG-tagged c-Myc fragments.

**2. Supplementary Tables**

**Table 1. Primer s**equences for qRT-PCR

| **Gene** | **species** | **Primer Sequence (5'→3')** |
| --- | --- | --- |
| c-Myc | mouse | Forward 5'-CCTCCACTCGGAAGGACTATC-3'  Reverse 5'-TTGTGTGTTCGCCTCTTGAC-3' |

**Table 2.** Primer sequences for nMS-PCR

| **Gene** | **species** | **Primer Sequence (5'→3')** |
| --- | --- | --- |
| outer primer | mouse | Forward 5'-AGGAAGAGAAAAATGTTGTTATTA-3'  Reverse 5'- AACAAAACCTAAATTACTCCTAAAC-3' |
| Methylation primer | mouse | Forward 5'- TAGTATTTGGGAGATTGAGGAATAC-3'  Reverse 5'- AAATAAAAAATCCTAAAACATCGAC-3' |
| Unmethylation primer | mouse | Forward 5'- GTATTTGGGAGATTGAGGAATATGA-3'  Reverse 5'- AAATAAAAAATCCTAAAACATCAAC-3' |
